# Nano-CT characterization of dentinal tubule occlusion in SDF-treated dentin

**DOI:** 10.1038/s41598-023-42805-8

**Published:** 2023-09-23

**Authors:** Matthias Menzel, Andreas Kiesow, Juliana Martins de Souza e Silva

**Affiliations:** Fraunhofer Institute for Microstructure of Materials and Systems IMWS, Halle (Saale), Germany

**Keywords:** Dental materials, Dental treatments, Biophysics

## Abstract

Dentin hypersensitivity is an oral health concern affecting a large percentage of the world's adult population. Occlusion of the exposed dentinal tubules is among the treatment options available, and silver diammine fluoride (SDF) is an occluding agent used for interrupting or dampening the stimulus of the dental pulp nerves that produce pain. In addition to dentin permeability testing, the evaluation of desensitizing agents occluding dentinal tubules strongly relies on microscopic techniques, such as scanning electron microscopy (SEM). Limitations of SEM are that it provides only surface images that lack detailed information on the depth of penetration and amount of material present within the treated specimen, and it is prone to sample preparation artifacts. Here, we present high-resolution X-ray computed tomography (nano-CT) as a potential method for investigating dentin specimens with occluded tubules. We studied human dentin treated with SDF as an exemplary dentinal occlusion treatment option. We evaluated the silver deposits formed on the dentin surface region near the dentinal tubules and in the tubular regions using cross-section SEM, Energy Dispersive X-ray (EDX) analysis, and nano-CT. The resulting images obtained by SEM and nano-CT had comparable resolutions, and both techniques produced images of the tubules' occlusion. Nano-CT provided three-dimensional images adequate to quantitate tubule size and orientation in space. Moreover, it enabled clear visualization of dentinal tubules in any virtual plane and estimation of the amount and depth of occluding material. Thus, nano-CT has the potential to be a valuable technique for evaluating the occluding effects of virtually any material applied to dentinal tubules, supporting deciding between the best occluding treatment options.

## Introduction

Silver diammine fluoride (SDF) solution is used for preventing and arresting carious lesions in adults and children^1–4^, and to reduce dentin hypersensitivity^5,6^, the short, sharp pain resulting from external stimulation^7,8^. The occlusion of dentinal tubules’ apertures by desensitizing agents is the primary strategy currently used by clinicians to prevent the pain^9–11^. In vitro models are usually used to understand the occlusion efficacy of desensitizing agents^12^, assessed by morphological analysis of precipitates formed in the dentinal tubules through microscopic methods^13–17^.

Those methods include Scanning Electron Microscopy (SEM)^18–20^, which can be combined with Energy Dispersive X-ray (EDX) analysis to provide surface morphological information, and the identity and ratio of chemical elements on and in the dentinal tubules^16,17,21^. SEM cross-section imaging provides additional information on occluding material penetration depth within the exposed imaged surface^15,22^. Different approaches can expose a cross-section of dentin specimens, such as fracturing the sample^18^, or using a Focused Ion Beam (FIB)^23,24^. These preparation techniques, however, are not free from artifacts. Fracturing the sample may add debris to the tubules that could be mistaken as occluding material, and FIB-SEM produces a three-dimensional image from serial two-dimensional SEM images^13,24^ in a process that is often challenging, time-consuming and requires post-acquisition image processing, which is limiting to most users^25^. Thus, the assessment of occlusion depth and the identification of different occluding materials in treated dentin specimens through morphological data can be difficult, either by limitations related to sample preparation or intrinsic to the microscopic imaging technique itself.

X-ray computed tomography (CT) is a cutting-edge imaging technology able to produce volumetric information that has been successfully applied to image small features in biomaterials^26^. Micro-computed tomography (micro-CT) was used to investigate the effect of SDF on carious lesions^27^ and on the enamel surface^28^. High-resolution CT (nano-CT) was already used to produce images of the dentin, giving details of the architecture of the dentinal tubules in different species at the nanometer scale^29–34^. Additionally, it can often access the distribution of the electron density, enabling quantitative analysis of the features observed in the specimens analyzed^29,34^. Nano-CT requires sample preparation that can create artifacts in the area where the specimen is processed, but the region of interest for imaging can remain unaffected and artifacts-free. To the best of our knowledge, nano-CT has not been used for the visualization of the occlusion of dentinal tubules after a treatment. Thus, here we show the potential of this technique for assessing dentinal tubule occlusion. We used two different SDF formulations to illustrate the ability of nano-CT to detect their occlusion efficacy and we discuss nano-CT benefits and disadvantages when compared to traditional microscopic methods.

## Materials and methods

### Samples analyzed

The samples were prepared as described previously^19^. Briefly, eighteen specimens of 4 mm × 4 mm dentin from human tooth roots were prepared to mimic exposed dentin after gingival recession. Human dentin specimens were prepared at Indiana University from human tooth roots. Ethical approval for use of the extracted teeth was given by the Indiana University Institutional Review Board (NSO 911-07). The specimens were polished and, to open their dentinal tubules, all specimens were immersed in a 17% EDTA solution (pH 7.4; Fisher Scientific) for 5 min, then rinsed with running deionized water for 5 s. They were then randomly allocated in equal amounts to three test groups (*n* = 6 for each group). Specimens in test group 1 were treated with one drop (c.a. 50 μL) of a standard commercial 38% SDF formulation (Advantage Arrest^®^, Elevate Oral Care LLC, with viscosity similar to water at room temperature) applied with a micro-brush. The same procedure was done in test group 2, in which the specimens were treated with an experimental SDF formulation (prepared by Elevate Oral Care LLC and based on Advantage Arrest^®^) that contains 38% SDF but with a higher viscosity (~ 30 cP at room temperature). In test group 3, specimens were treated with a placebo solution, free from fluoride or silver, that was freshly prepared and certified by an FDA-regulated laboratory independent of the investigators. The solutions applied onto the specimens remained on the exposed dentin surfaces undisturbed for 1 min, then rinsed with running deionized water for 5 s. The specimens were then immersed in artificial saliva (2.2 g L^−1^ gastric mucin, 0.381 g L ^-1^ NaCl, 0.213 g L^−1^ CaCl_2_·H_2_O, 0.738 g L^−1^ KH_2_PO_4_, 1.114 g L^−1^ KCl, pH 7.0; Fisher Scientific) for 2 h^35^, rinsed again with running deionized water for 5 s and the vials with excess moisture were sealed and shipped to the imaging facility.

### SEM imaging

A total of four specimens from each of the three groups were prepared as illustrated in the supplementary information Fig. S1 for surface and cross-section analysis of the dentinal tubules. Sample pieces were deposited onto carbon tape, sputtered with a palladium layer of a few nanometers, and imaged in a SEM (Quanta3D FEG Dual-Beam—FEI Company) working at an acceleration voltage of 10 kV. EDX was performed using an Oxford Xplore EDX-Detector (Oxford Instruments) (Tables S1–S3). A droplet of each solution (placebo, SDF commercial and experimental formulations) was allowed to dry over a SEM stub that was covered with carbon tape. The residues of each solution were analyzed by EDX (Tables S4–S6).

### Nano-CT imaging

Two specimens from each of the three groups were prepared as illustrated in supplementary information Fig. S1b for volumetric imaging of the dentinal tubules. Samples were laser machined in the shape of cones with 300 µm height and a tip of ca. 50 µm using a laser preparation tool (microPREP™, 3D-Micromac, Germany)^36^. Each cone was then glued onto the top of a metallic pin and imaged using ZEISS Xradia 810 Ultra nanoscale X-ray microscope operating with a chromium X-ray source (5.4 keV). Samples were imaged using absorption-contrast. A total of 901 projections were obtained over 180°, a camera binning of 2 (128 nm of final isotropic voxel size) and exposure time of 30 s per projection. Image reconstruction was performed by a filtered back-projection algorithm using the software XMReconstructor integrated into the device. Volumetric data was visualized either in Avizo (Thermo Fisher Scientific, version 2021.2) or VGStudio (version 2022.2). For the estimation of silver within the tubules, data were processed in Avizo, first matching the contrast between the two datasets (dentin treated either with commercial or experimental formulation) to assure that the signal from silver was comparable between datasets. Then, a median filter was applied for image correction, followed by segmentation using interactive threshold, with the threshold defined by the clear boarders seen in the tomograms (Figs. S2 and S3). After segmentation, a smaller volume of the datasets (300 × 300 × 875) was selected to remove the boarders and empty areas around the specimens. The volume fraction of silver signal for each xy-slice (300 × 300) in each dataset of specimens treated with SDF was estimated and it corresponds to the ratio of the number of pixels corresponding to silver to the total number of pixels in the slice. The estimation of silver percentage in the samples was obtained by the ratio of the volume occupied by the voxels related to silver over the volume occupied by the voxels related to tubules.

## Results

**Figure 1:**
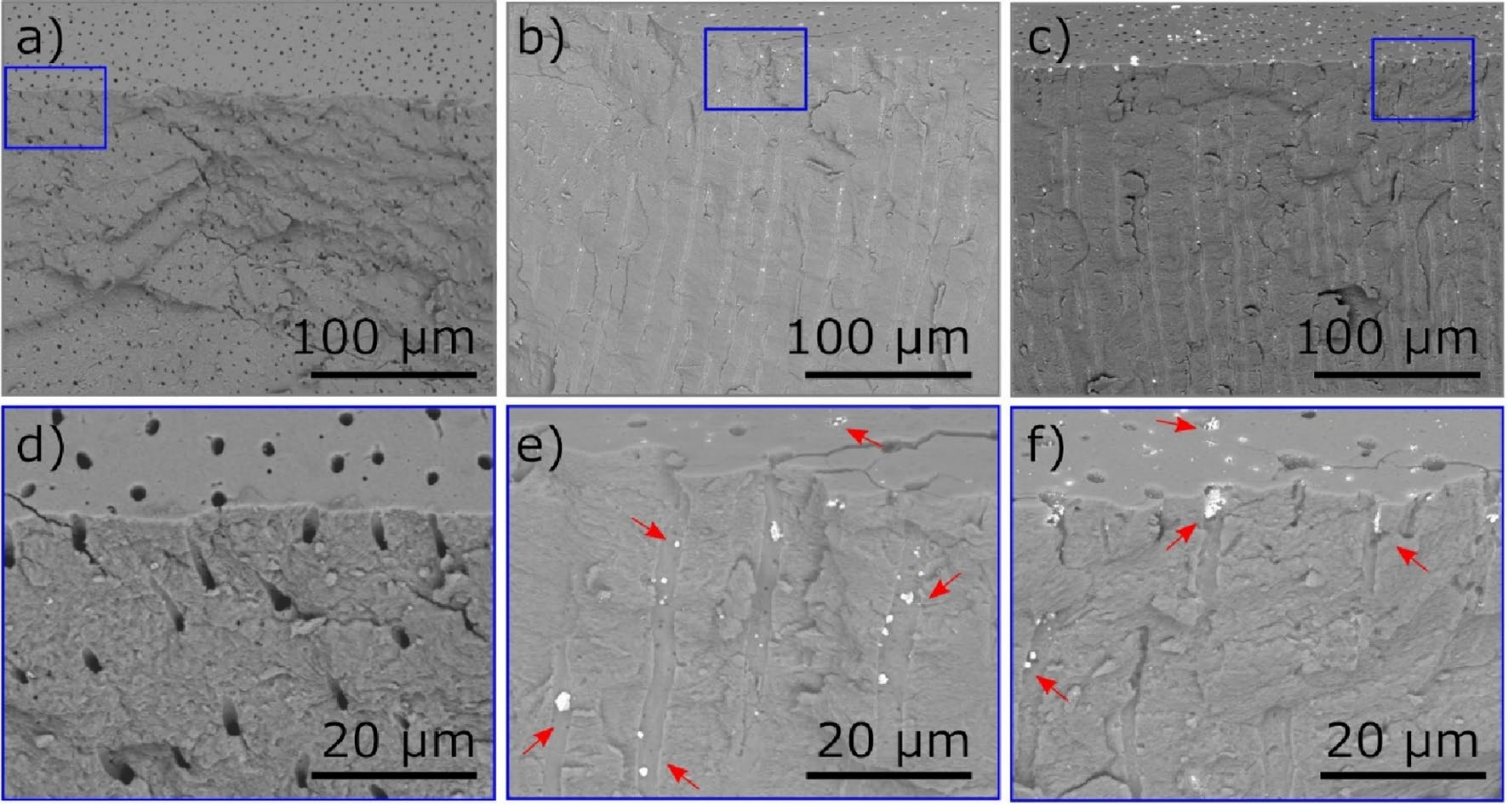
SEM images of samples treated with (**a**) placebo; (**b**) commercial SDF formulation; and (**c**) experimental SDF formulation, (**d**–**f**) are the enlarged highlighted areas in (**a**–**c**), respectively. Red arrows indicate a few regions with bright material accumulation.

To image the dentinal tubules treated with different SDF formulations, we first used the traditional method of cross-section SEM (supplementary information Fig. S1a). The SEM images of the cross-sections of the specimen treated with placebo (Fig. 1a) show completely open and unobstructed tubules, as expected. The dentin of specimens treated with two different SDF formulations—the commercial (Fig. 1b) and the newly developed (Fig. 1c)—show deposits on the dentin surface mainly occluding the dentinal tubules. The enlarged images of the treated specimens (Fig. 1d–f) show that the main difference among them is the presence of bright deposits of different sizes and at variable depths within the tubules of the specimens treated with SDF (red arrows in Fig. 1e,f).

**Figure 2:**
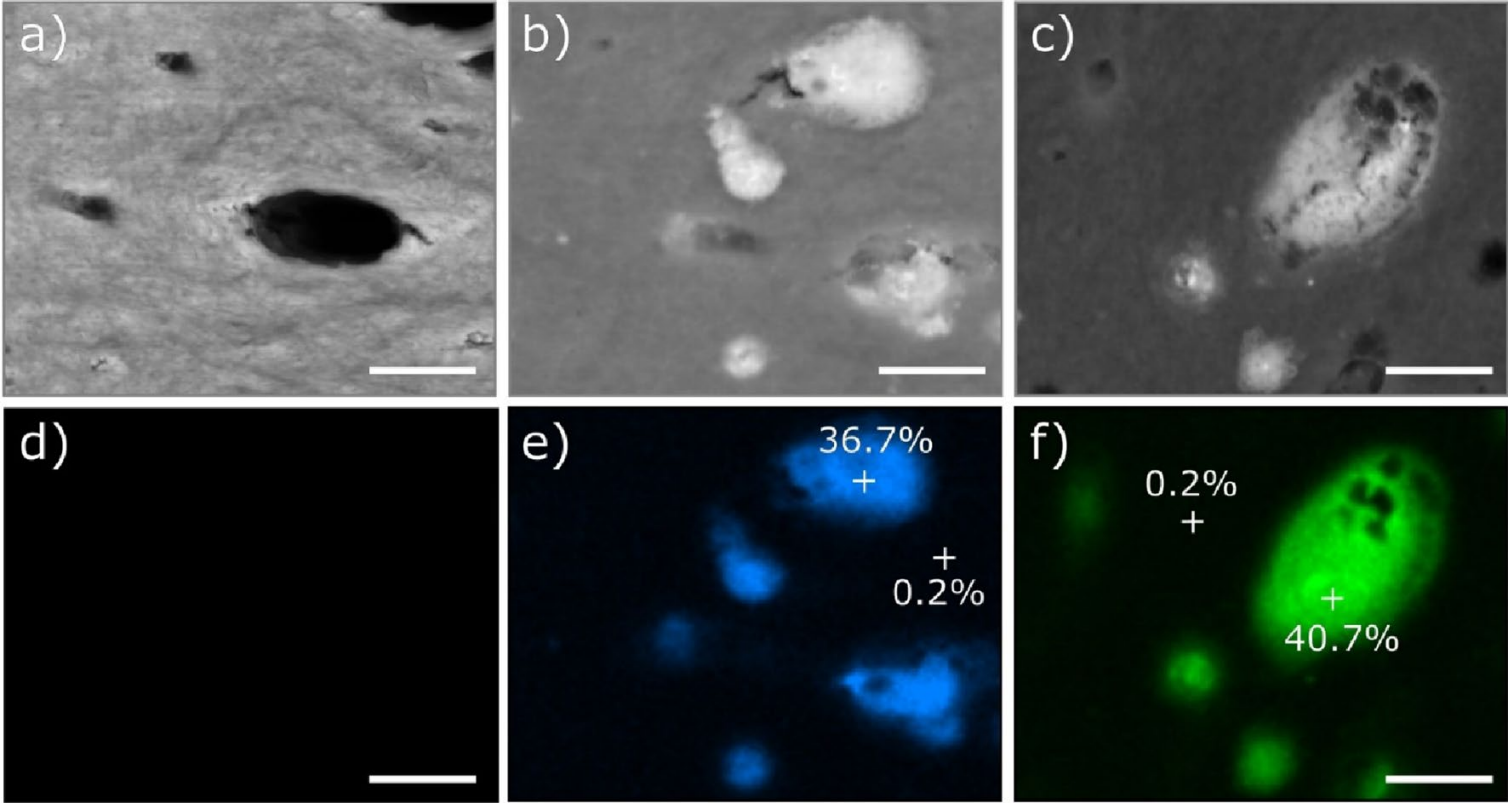
EDX elemental mapping image of the dentin surface and occluded tubules. SEM images of samples treated with (**a**) placebo; (**b**) commercial SDF formulation; and (**c**) experimental SDF formulation, and below (**d**–**f**) the corresponding silver signal detected by EDX. Scale bar: 100 µm. Silver amount (atomic percentage) is given for the areas marked with a cross in (**e**,**f**). Scale bar: 2.5 µm.

We used EDX combined to SEM to create a map of the distribution of the elements present on and near the surface of the specimens. The composition of the bright spots measured by EDX elemental mapping corresponds mainly to silver (Fig. 2 and supplementary information Tables S2 and S3) in agreement with the SDF solutions composition (Tables S4–S6). Palladium detected results from the sputtering step in the sample preparation process. The dentin surface of the untreated sample (Fig. 2a,d), which does not show any bright spots, has a composition that is consistent with a typical mineral-density profile (Table S1)^37^. The elemental mapping of the dentin surfaces treated with SDF (Fig. 2b,c,e,f) shows that the bright spots have a high silver amount and are located mainly on top of the tubules, occluding them. The silver amount measured on the top of the occluded tubules is slightly different between the samples treated with the two SDF formulations (commercial: 36.7% vs. experimental, 40.7%, respectively in Fig. 2e,f, and Tables S2 and S3).

**Figure 3:**
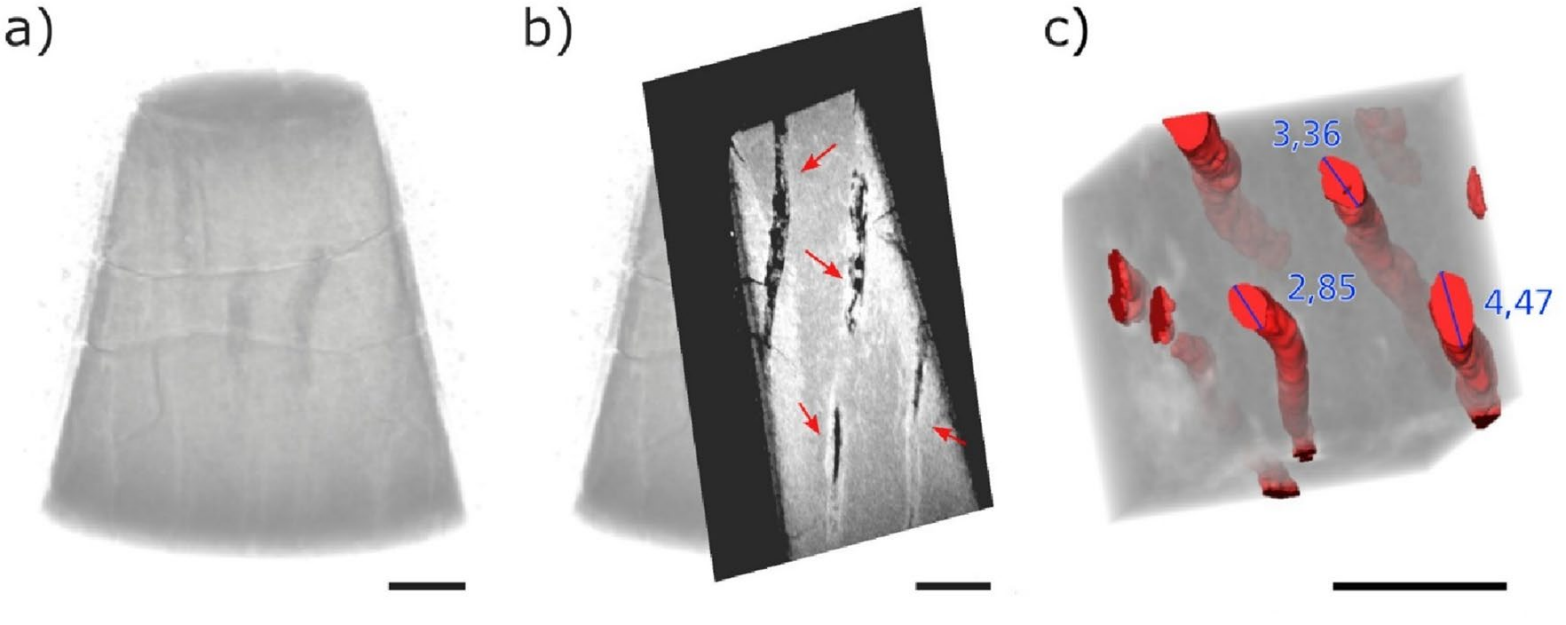
(**a**) Volumetric reconstruction of a conical-shaped dentin specimen treated with placebo formulation, (**b**) virtual frontal cut, with red arrows indicating the tubules, and (**c**) 3D reconstruction of a smaller volume of interest within the specimen, showing a few tubules (red) separated from the rest of the sample (in transparent grey tone). Numbers give the larger diameter of a few tubules marked in blue. Scale bar: 10 µm.

To enable the visualization of the tubules located inside the specimens and not exposed by the cross sectioning, we used high-resolution X-ray computed tomography (nano-CT). The nano-CT 3D image of the conical specimen treated with placebo solution (Fig. 3a, and Movie S1) shows tubules in darker tone and some horizontal cracks related to the sample preparation procedure (Fig. S1b). A virtual front cut of the 3D image shows the empty tubules in black oriented vertically in the image (red arrows in Fig. 3b). We further analyzed a smaller volume of the dentin and virtually separated the tubules from the rest of the specimen (tubules pseudo-colored in red in Fig. 3c). The tubules’ diameter estimated from these images varied between 2.9 and 4.5 µm.

**Figure 4:**
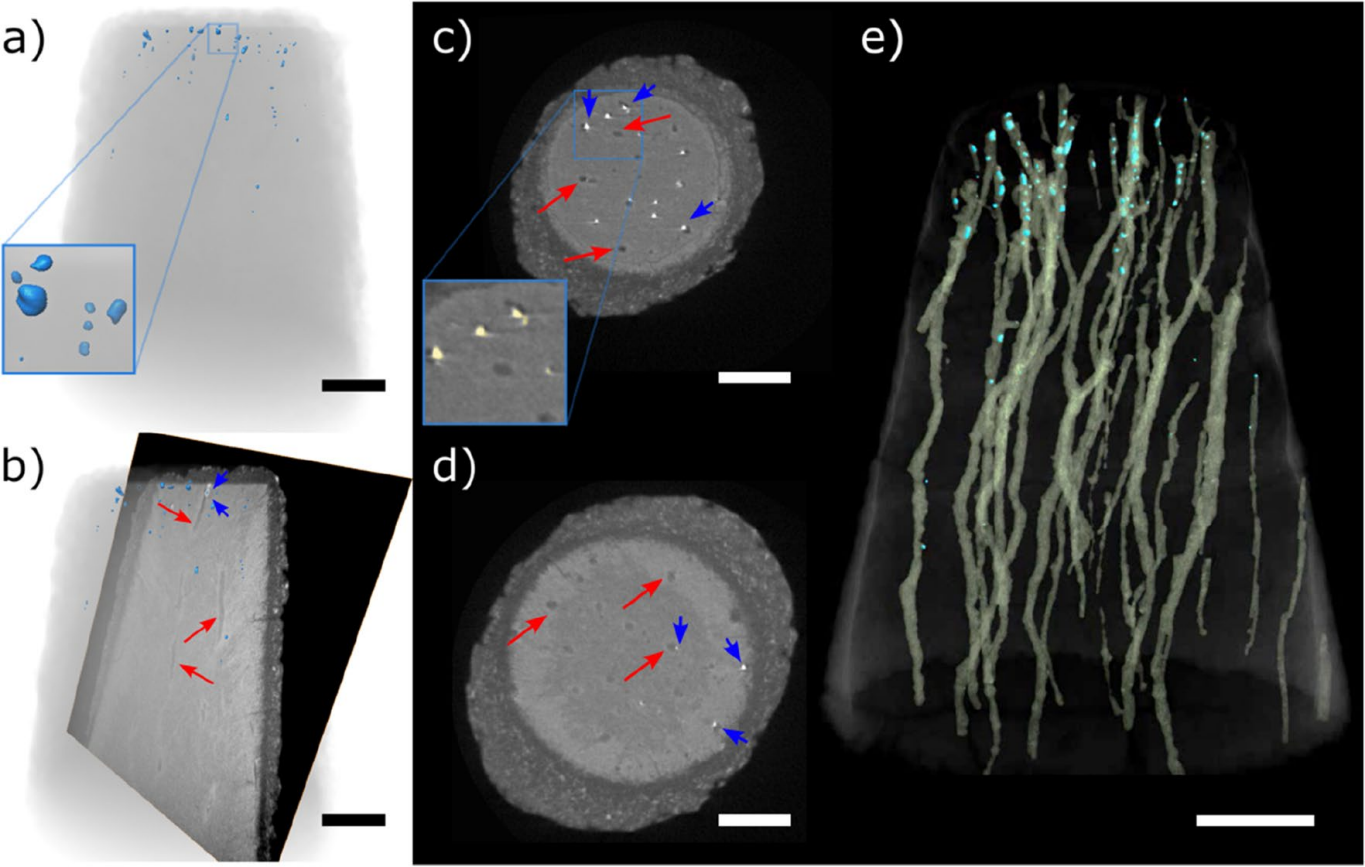
(**a**) Volumetric reconstruction of a conical-shaped dentin specimen treated with commercial SDF formulation (dentin in transparent grey and silver agglomerates in blue). Enlarged part of the volumetric image within the blue square has 5 × 5 µm^2^. (**b**) Virtual frontal cut of the specimen volume, (**c**,**d**) are horizontal virtual slices (respectively 3.0 and 9.5 µm from the top of the specimen), with a zoomed area with segmented particles within a blue square of 20 × 20 µm^2^, and (**e**) volumetric reconstruction of the dentin specimen in which dentin is shown in transparent tone to highlight the tubules (pseudo-colored yellow) and the silver agglomerates (pseudo-colored in blue). Scale bars: 10 µm. Red arrows indicate tubules and blue arrows show silver agglomerates.

**Figure 5:**
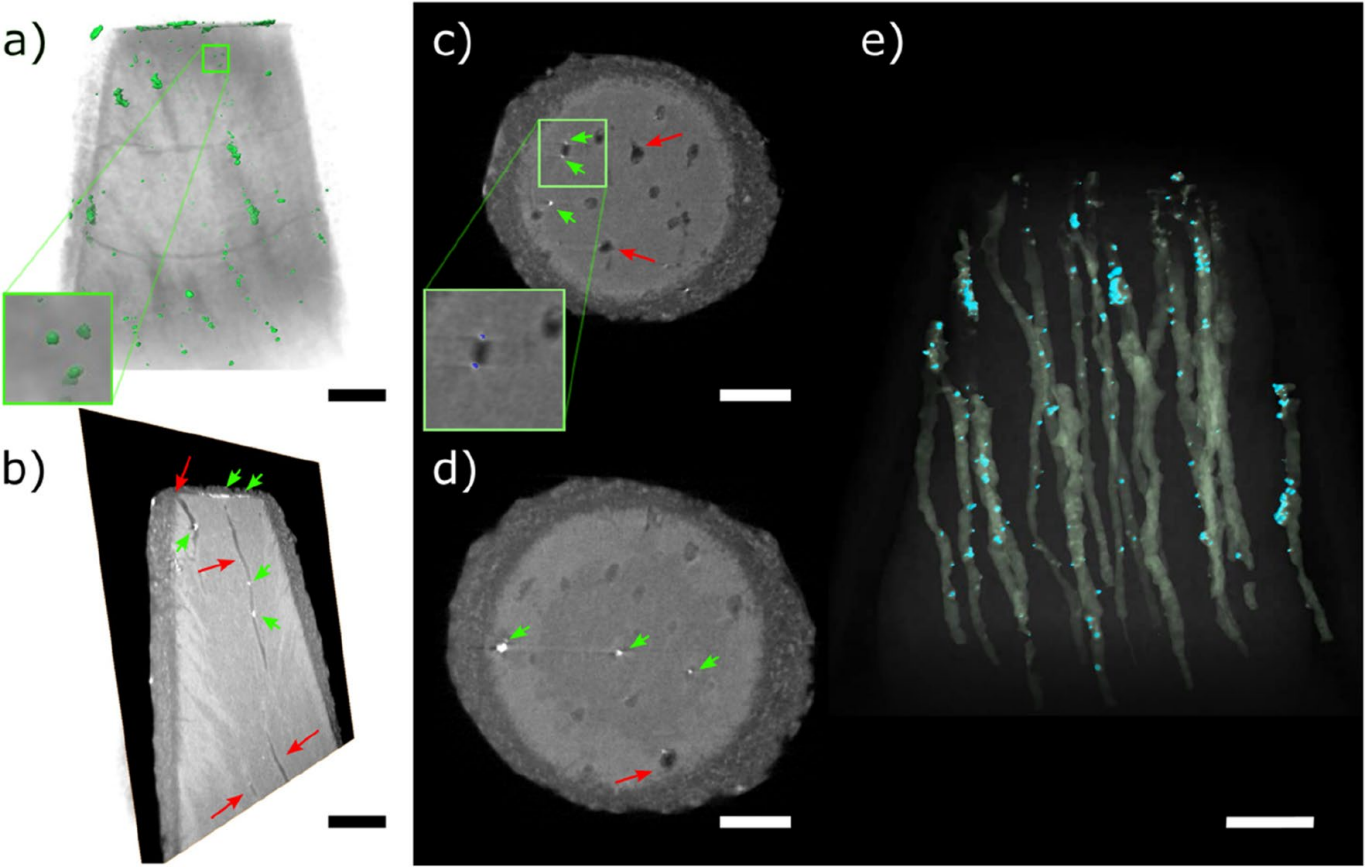
(**a**) Volumetric reconstruction of a conical-shaped dentin specimen treated with experimental SDF formulation (dentin in transparent grey and silver agglomerates in green), showing an enlarged part of the volumetric image within a green square of 5 × 5 µm^2^. (**b**) Virtual frontal cut of the specimen volume, (**c**,**d**) are horizontal virtual slices (respectively 3.0 and 9.5 µm from the top of the specimen) with a zoomed area with segmented particles within a green square of 20 × 20 µm^2^, and (**e**) volumetric reconstruction of the dentin specimen in which dentin is shown in transparent tone to highlight the tubules (pseudo-colored with a yellowish tone) and the silver agglomerates (pseudo-colored in blue). Scale bars: 10 µm. Red arrows indicate tubules and green arrows show silver agglomerates.

Different than the placebo formulation-treated sample, the volumetric images of SDF-treated samples (Figs. 4 and 5, and Movies S2 and S3) show tubules occluded by bright spots that are attributed to silver deposits (Fig. S2 and S3). The volumetric image (Fig. 4a) of the dentin specimen and the silver agglomerates virtually segmented from the rest of the sample (pseudo-colored in blue in Fig. 4a) show that the agglomerates are mainly located close to the top, which is the application surface. The 3D image can be virtually cut to show the depth of penetration of these agglomerates. The virtual frontal cut of the specimen treated with commercial SDF formulation (Fig. 4b) shows an image with information comparable to that obtained by cross-section SEM, with tubules containing bright particles, mainly located on the top part. That is also shown by the horizontal virtual slices at a depth of 2.3 and 9.5 µm from the top of the specimen, respectively, with more (blue arrows in Fig. 4c) or less agglomerates (blue arrows in Fig. 4d). The volumetric image also enables the visualization of single tubules and the silver within it (Fig. 4e). Though silver is still detected at 20 µm of depth, most silver agglomerates are detected in the first 5 µm from the SDF application surface (top of the specimen). From the volumetric image, we estimated the volume occupied by the tubules (761 µm^3^) and the volume occupied by the silver-containing particles (4.8 µm^3^). This information enabled the estimation of the silver percentage in relation to the tubules volume, which is equal to 0.63% for this specimen.

The volumetric reconstruction of the sample treated with the experimental SDF formulation shows silver agglomeration in the tubules (Fig. 5a), besides some horizontal cracks resulting from the sample preparation protocol (Fig. S1b). The virtual frontal cut (Fig. 5b) shows tubules containing bright particles, which we attribute to silver from the SDF formulation. The horizontal virtual cuts (Fig. 5c,d) illustrate the depth of penetration of the silver deposits, showing that most silver particles are present closer to the top of the specimen. The volumetric dataset enables the virtual separation of the tubules and silver agglomerates from the dentin (Fig. 5e). From the volumetric image, we estimated the volume occupied by the tubules (410 µm^3^) and the volume occupied by the silver-containing particles (6.9 µm^3^). Assuming that the particles are composed purely by silver, this information enabled the estimation of the silver percentage in relation to the tubules volume, which is equal to 1.68% for this specimen.

**Figure 6:**
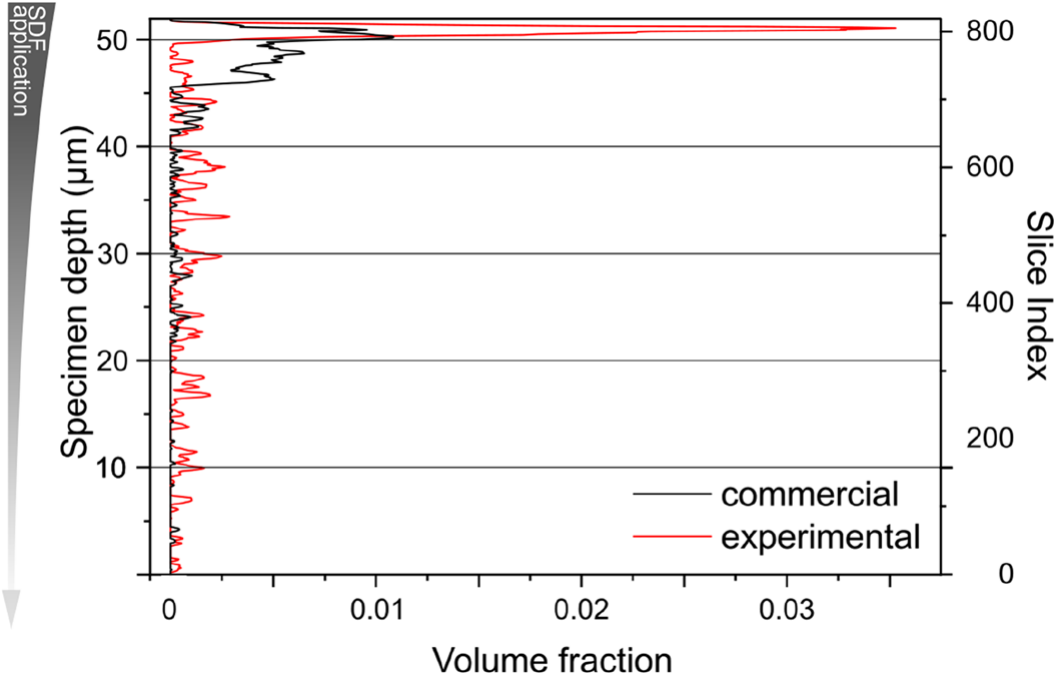
Depth of silver penetration for commercial (black curve) and experimental (red curve) SDF formulations. The amount of silver is expressed in terms of volume fraction, which is the ratio of the pixels assigned to silver and all the pixels in each slice, from bottom of the specimen (slice 0) to the top.

We show in Fig. 6 an example of how the penetration depth can be quantified and used to compare different formulations. We used the change in the amount of silver from the application point (top of the specimen) downwards to compare the penetration depth of SDF between two samples, one treated with commercial SDF formulation and the other with the experimental counterpart. Though the highest amount of silver is found on the top part for both formulations, a difference is observed between them. Most of silver is located close to the application point for the commercial formulation, in the top 6 µm, and smaller amounts of silver are found up to 30 µm deeper into the specimen. Almost no more silver is found further than 30 µm of depth. For the specimen treated with the experimental formulation, the highest concentration of silver is found within the top 2 µm and smaller amounts of silver are found distributed within the tubules up to 50 µm of depth.

## Discussion

In this work, we imaged dentin samples treated with different SDF formulations using cross-section SEM, EDX and nano-CT. The images obtained by SEM and nano-CT show the dentinal tubules in detail. They also show that the SDF treatment of the dentin samples resulted in the occlusion of a few tubules by silver particles, not wires, as observed by others^38^. In part the difference may be because the teeth studied in this earlier work had large natural caries lesions with greater destruction of the tubule architecture than the sound dentin treated with EDTA in this study. With EDX analysis (Tables S1, S2 and S3), characteristic X-rays of the elements found on and near the surface of the specimen are detected. It our study, it allowed the creation of a map of the distribution of silver on the dentin surface.

Sample preparation through cross-sectioning and SEM imaging (Fig. S1a) took roughly 2 h in this study to obtain the images shown here. For nano-CT, we cut the sample in a conic shape in a procedure that takes 30–60 min. Imaging each sample with nano-CT took 8 h. Image processing for the separation of the parts composing the image (dentin, tubules filled with air, containing or not silver deposits), in a process known as image segmentation, varies according to the experience of the analyst processing the images. Here, the process took roughly 2 h per sample. After segmentation, estimation of tubules diameter and silver penetration depth is a matter of seconds.

Though SEM is a technique that requires less time for sample preparation, imaging, and image processing, the cross-section images obtained are limited to showing only a few tubules in two-dimensional images of one sectioned plane. Sample preparation requires sample fracturing or cutting, a process prone to artifacts or the formation of a section that is inadequate for visualizing the structures of interest and can be inconclusive^39,40^. In comparison, nano-CT produces three-dimensional representations of the samples that can be virtually cut in any plane without the need to physically section the specimen and, thus, does not generate sectioning artifacts. Nano-CT enabled the three-dimensional visualization of the tubules within the dentin of the placebo-treated sample and the tubules’ diameter estimation. While the cross-section SEM images give an impression of the distribution of silver deposits within the tubules of the SDF-treated specimens, it lacks the volumetric information on the depth of penetration of silver for a larger volume. Besides giving the penetration depth of silver in the images, nano-CT also enables the estimation of silver volume percentage in the entire specimen, which is adequate for quantitative and statistic studies. Of course, FIB-SEM also provides three-dimensional images of dentin. However, the larger the volume analyzed, the more challenging and time-consuming image acquisition and processing are.

As an exemplary analysis, we have shown the dentin-tubule occlusion and penetration behavior for two different SDF treatments. The dentin treated with the standard SDF formulation had a lower amount of silver located mostly close to the surface. The specimen treated with the new and more viscous SDF formulation had more silver detected deeper into the tubules. Though the higher viscosity would explain these differences as it would enable the application of the SDF in a more controlled way, the analysis of more specimens would be necessary to produce statistically relevant data.

With nano-CT, we have been able to visually demonstrate the effective occlusion of SDF. While other filling agents like silica, hydroxyapatite, oxalates, and carbonates^8,41–43^ may not absorb X-rays as strongly as SDF, they can still be imaged using different energies to provide quantitative information on their occluding capabilities and penetration depth. It is crucial, though, to differentiate these materials from natural irregularities or noise in imaging. Nano-CT is a comprehensive technique that can investigate the interaction between these materials and dentin after comparative treatments. It is particularly useful when dentin treatment strategies involve materials with high contrast, providing clear advantages over SEM in studies that require statistical analysis of occluding particles. However, precise imaging methods like nano-CT and SEM have the limitation of only analyzing a small area of the specimen, which may not provide reliable representation of the entire sample treatment. Therefore, analyzing multiple specimens and using complementary techniques is necessary to obtain relevant statistical data.

## Conclusion

In this work, we studied dentin samples treated with two different SDF formulations and using two types of microscopy: (cross-section) SEM combined with EDX, and nano-CT. We evaluated silver deposits formed on the dentin surface near the dentinal tubules and in the tubular regions. Our findings show that nano-CT is a useful method for studying dentin. It produces high-resolution 3D images that enable clear visualization of dentinal tubule detail, showing features as small as a few hundred nanometers. Though we used the widely adopted SDF as an example, nano-CT can be used to image all types of materials that produce X-ray contrast against the dentin composition. Our data illustrates that nano-CT is a powerful technique for non-destructive imaging within the volume analyzed, enabling detailed characterization of tubule obstruction. Moreover, it can produce volumetric quantitative data when statistical analysis is of interest, such as when comparing different dentin treatments. Nano-CT enables quantitative evaluation of tubule size and orientation in space, allowing estimation of the volume occupied by occluding particles within the tubules. Thus, nano-CT has the potential to be one alternative methodology for the evaluation of tubular occlusion. We anticipate that it will benefit the evaluation of the occluding effects of virtually any material applied to dentinal tubules, showing the penetration depth of the material within an entire volume of tens of micrometers, thus aiding in the development of more effective interventions for preventing dentin hypersensitivity.

### Supplementary Information

Supplementary Video 1.

Supplementary Video 2.

Supplementary Video 3.

Supplementary Information.

## Data Availability

Data is available from the corresponding authors upon request.
