# Supplementary material for: Nano-CT characterization of dentinal tubule occlusion in SDF-treated dentin
Source: Sci Rep. 2023 Sep 23;13:15895. doi: 10.1038/s41598-023-42805-8 (PMC10517917; doi:10.1038/s41598-023-42805-8)
Supplement: Supplementary file 4 — Supplementary Information. [file 41598_2023_42805_MOESM4_ESM.pdf]

Supplementary material

Nano-CT characterization of dentinal tubule occlusion in SDF-treated dentin

Matthias Menzel[1], Andreas Kiesow[1], Juliana Martins de Souza e Silva[1,*]

[1] Fraunhofer Institute for Microstructure of Materials and Systems IMWS, Halle (Saale), Germany

*juliana.martins-schalinski@imws.fraunhofer.de

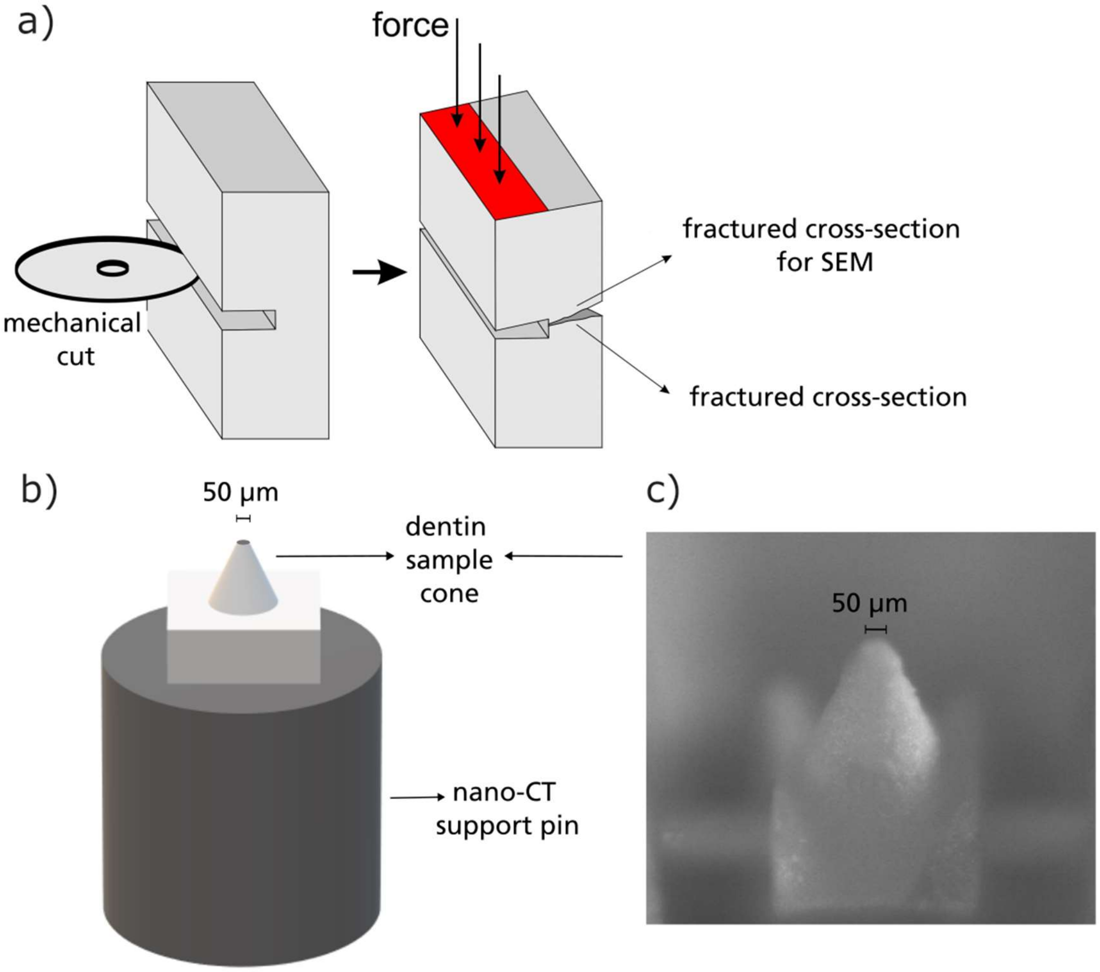

Figure S1. a) Schematic illustration of the fracture preparation of the dentin specimens for cross-section SEM. The specimens were pre-sawed on the backside (opposite site of treatment) to ensure crack initiation. b) Illustration of nano-CT cone preparation, with an optical microscopy image of a real specimen in c).

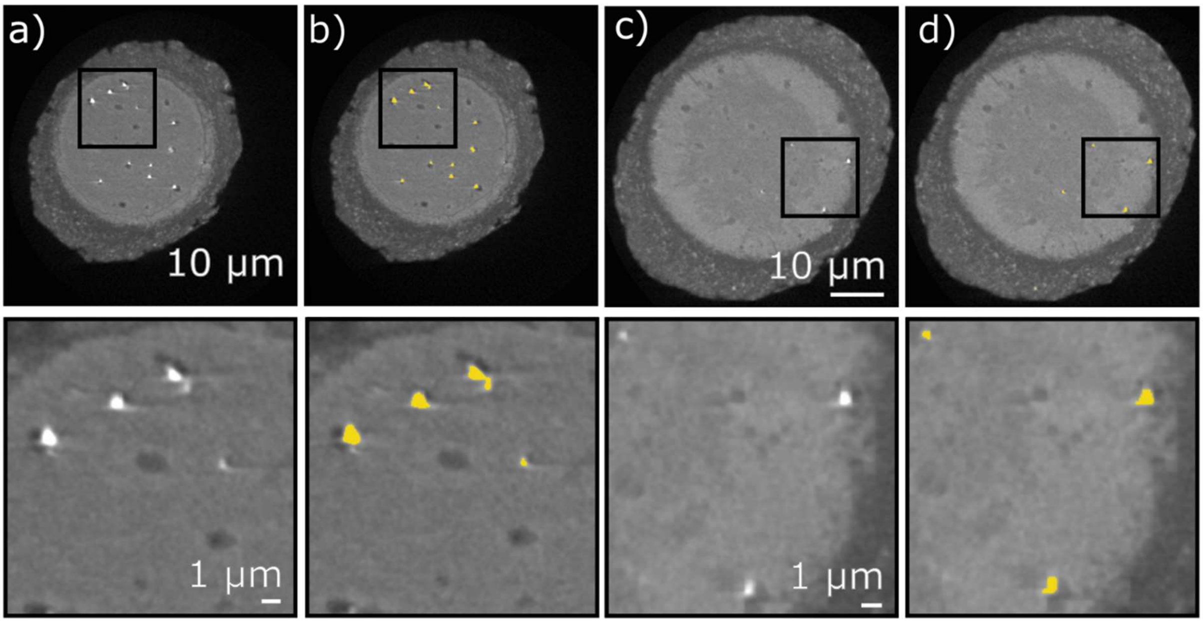

Figure S2. Tomograms obtained by nano-CT dentin specimen treated with the commercial SDF formulation. a) Tomogram 3 µm distant from the application position (top of the specimen), b) same tomogram as a) after segmentation of the silver-containing particles (yellow), c) tomogram 9,5 µm distant from the application position, d) same tomogram as c) after segmentation of the silver-containing particles (yellow). Enlarged region within black square immediately below each tomogram.

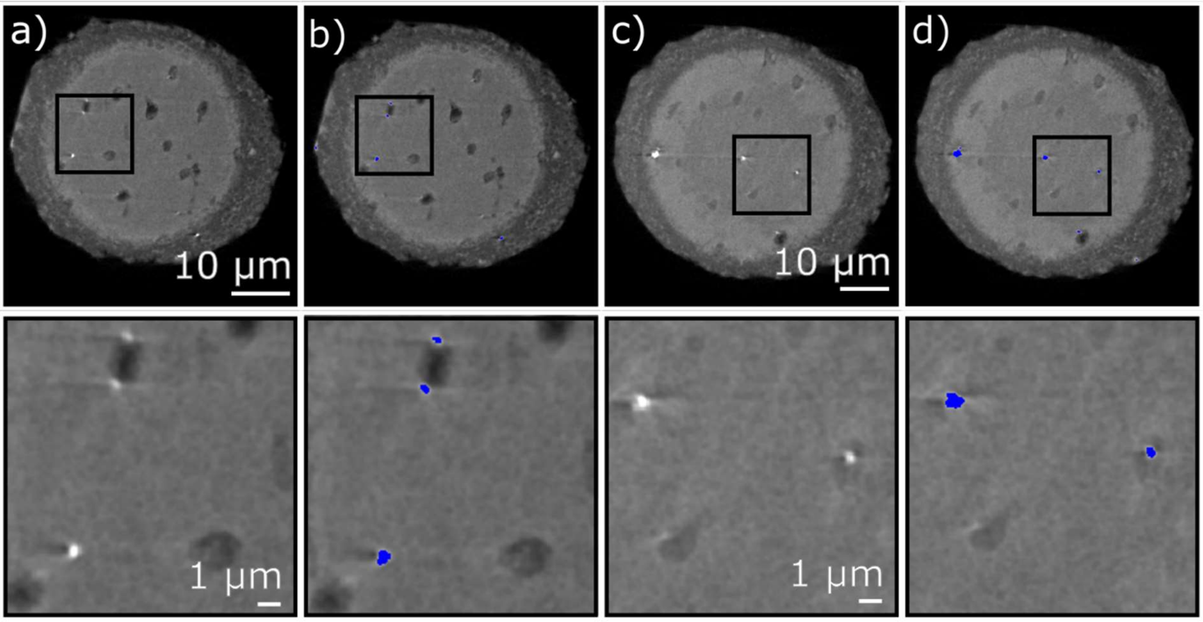

Figure S3. Tomograms obtained by nano-CT dentin specimen treated with the experimental SDF formulation. a) Tomogram 3 µm distant from the application position (top of the specimen), b) same tomogram as a) after segmentation of the silver-containing particles (blue), c) tomogram 9,5 µm distant from the application position, d) same tomogram as c) after segmentation of the silver-containing particles (blue). Enlarged region within black square immediately below each tomogram.

Table S1. Chemical composition of dentin surface treated with placebo obtained by EDX.

Sample	Atomic %										
	C	N	O	F	Na	Mg	P	Ca	Pd	Ag	Total
Dentin	27.50	9.70	39.20	0.00	0.40	0.71	8.31	13.50	0.67	0.01	100.00
Tubuli	25.22	7.94	44.99	0.00	0.42	0.64	7.70	12.37	0.70	0.00	100.00

Table S2. Chemical composition of dentin surface treated with commercial SDF formulation obtained by EDX.

Sample	Atomic %										
	C	N	O	F	Na	Mg	P	Ca	Pd	Ag	Total
Dentin	24.44	8.89	39.37	0.27	0.42	0.74	9.57	15.46	0.67	0.16	100.00
Tubuli	37.52	13.03	4.96	0.43	0.00	0.20	0.83	1.23	5.10	36.69	100.00

Table S3. Chemical composition of dentin surface treated with experimental SDF formulation obtained by EDX.

Sample	Atomic %										
	C	N	O	F	Na	Mg	P	Ca	Pd	Ag	Total
Dentin	17.22	8.55	35.82	0.20	0.32	0.90	13.58	22.43	0.82	0.15	100.00
Tubuli	30.24	15.36	5.81	0.00	0.00	0.16	1.08	1.27	5.34	40.74	100.00

Table S4. Chemical composition of placebo solution obtained by EDX.

Atomic %										
C	N	O	F	Na	Mg	P	Ca	Pd	Ag	Total
89.36	0.00	8.92	0.16	1.50	0.01	0.01	0.01	0.00	0.00	100.00

Table S5. Chemical composition of commercial SDF formulation obtained by EDX.

Atomic %										
C	N	O	F	Na	Mg	P	Ca	Pd	Ag	Total
1.74	0.3	0.67	6.66	0.04	0.00	0.01	0.00	0.00	90.49	100.00

Table S6. Chemical composition of experimental SDF formulation obtained by EDX.

Atomic %										
C	N	O	F	Na	Mg	P	Ca	Pd	Ag	Total
2.01	0.00	0.71	9.14	0.00	0.00	0.02	0.00	0.00	88.06	100.00

Supplementary movies legends:

movie_1_placebo.wmv. Volumetric reconstruction of a conical-shaped dentin specimen treated with placebo formulation.

movie_2_commercial.wmv. Volumetric reconstruction of a conical-shaped dentin specimen treated with commercial formulation.

movie_3_experimental.wmv. Volumetric reconstruction of a conical-shaped dentin specimen treated with experimental formulation.
